# The Effect of Treadmill Training Pre-Exercise on Glutamate Receptor Expression in Rats after Cerebral Ischemia

**DOI:** 10.3390/ijms11072658

**Published:** 2010-07-07

**Authors:** Feng Zhang, Jie Jia, Yi Wu, Yongshan Hu, Yang Wang

**Affiliations:** 1 Department of Rehabilitation, Huashan Hospital, Fudan University, Shanghai, China; 2 Department of Anatomy and Embryology, Fudan University, Shanghai, China; E-Mail: zjk20019@126.com

**Keywords:** *mGluR5*, *NR2B*, real-time PCR, brain ischemic tolerance, ischemic preconditioning

## Abstract

Physical exercise has been demonstrated to be neuroprotective in both clinical and laboratory settings. However, the exact mechanism underlying this effect is unclear. Our study aimed to investigate whether pre-ischemic treadmill training could serve as a form of ischemic preconditioning in a rat model undergoing middle cerebral artery occlusion (MCAO). Thirty-six rats were divided into three groups: a sham control group, a non-exercise with operation group and an exercise with operation group. After treadmill training, ischemia was induced by occluding the MCA for 2 h, followed by reperfusion. Half of the rats in each group were sacrificed for mRNA detection of *mGluR5* and *NR2B* 80 min after occlusion. The remaining animals were evaluated for neurological deficits by behavioral scoring and then decapitated to assess the infarct volume. The mRNA expression of *mGluR5* and *NR2B* was detected by real-time PCR. The results suggest that pre-ischemic treadmill training may induce brain ischemic tolerance by reducing the mRNA levels of *mGluR5* and *NR2B*, and thus, the results indicate that physical exercise might be an effective method to establish ischemic preconditioning.

## 1. Introduction

Ischemic stroke causes devastating complications that result in a significant burden for the entire society [1]. Unfortunately, there are no effective strategies to successfully eliminate such complications, so greater attention should be given to the prevention of stroke. Accumulating evidence has indicated that physical exercise exerts beneficial effects on stroke injuries, including decreases in cerebral infarction and edema, promotion of higher survival rates and amelioration of inflammatory injuries [2–5]. The brain’s ability to develop a tolerance to subsequent ischemic injury after a moderate stimulus is known as the brain ischemic tolerance (BIT) phenomenon [6]. Several kinds of preconditioning that promote resistance to ischemic stroke have been studied, including ischemia [6], hypoxia [7] and anoxia [8]. However, these aforementioned pretreatments might not be easily applied as clinical intervention in individuals with risk factors for stroke. Several studies aiming to identify easily applied pretreatments that induce resistance to cerebral ischemia have suggested that pre-exercise with treadmill training may reduce risk factors for stroke [3,5,9].

Excessive release of glutamate after a stroke has been shown to lead to neuronal excitotoxicity and subsequent brain damage [10,11]. Glutamate receptor antagonists, such as those that target *NR2B* and *mGluR5,* have been shown to reduce brain damage after stroke [12,13]. Likewise, we previously showed that pre-ischemic treadmill training can down-regulate the excessive release of glutamate after ischemic stroke [14]. Therefore, we sought to explore whether pre-exercise with treadmill training directly affected glutamate receptors to clarify the mechanism underlying the neuroprotective effects of physical exercise. The striatum is known to be one of the core regions that is vulnerable to ischemic insult and enriched in glutamatergic neurons. According to our recently published paper [15], pre-ischemic training could influence this region by regulating the mGluR1. Furthermore, in our previous study, we found that the level of glutamate in the striatum increased to its peak value nearly 80 min after the occlusion of the middle cerebral artery (MCA) [14]. Therefore, we quantified the mRNA levels of NR2B and mGluR5 in the striatum by real-time PCR at this time point. Our results indicate that treadmill training down-regulated the expression of NR2B and mGluR5, possibly contributing to the brain ischemic tolerance induced by pre-exercise with treadmill training.

## 2. Results and Discussion

### 2.1. Physiological Variables

Animals were divided into a sham group, an exercise with operation group and a non-exercise with operation group. There were no significant differences observed in paO2 (partial pressure of oxygen in arterial blood), paCO2 (partial pressure of carbon dioxide in artery), or pH (hydrogen ion concentration) values between the sham, non-exercise with stroke and exercise with stroke groups before and after MCAO (Table 1).

### 2.2. Relative NR2B and mGluR5 mRNA Expression Levels among the Three Groups

Extracts from the striatum of the treated rats and sham controls (from rats decapitated 80 min after occlusion) were subjected to quantitative real-time PCR analysis.

**Figure 1.**
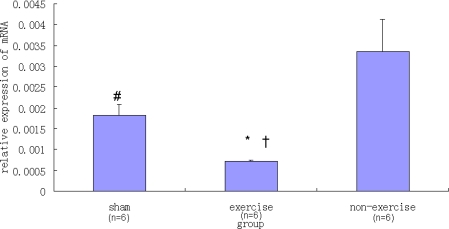
Real-time PCR analysis of relative *NR2B* mRNA expression levels in the striatum. Data are mean ± S.E.M (n = 6). There was significant difference (F_(2,15)_ = 16.24, P = 0.001) among the three groups studied. Signs indicate statistically significant difference (P < 0.05) in *NR2B* expression level between: the exercise with operation group and the sham control group ^*^ or the non-exercise with operation group ^†^; between the non-exercise group and the sham group ^#^.

*NR2B* expression was significantly lower in the exercise group compared to the sham group and the non-exercise group (P < 0.05), as indicated in Figure 1. There was also a significant difference in the relative *NR2B* expression between the non-exercise group and the sham group (P < 0.05).

**Figure 2.**
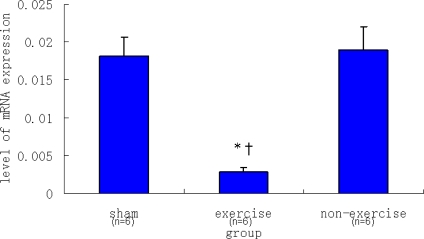
Real-time PCR analysis of relative *mGluR5* mRNA expression levels in the striatum. Data are mean ± S.E.M (n = 6). Signs indicate statistically significant difference (P < 0.05) in *mGluR5* expression level between the exercise with operation group and the sham control group ^*^ or the non-exercise with operation group ^†^. There was no significant difference in relative to *mGluR5* expression between the non-exercise group and the sham group (P > 0.05).

In regard to *mGluR5*, there was no significant difference in *mGluR5* expression between the non-exercise group and the sham group (P > 0.05). However, *mGluR5* expression was significantly decreased in the exercise group compared to the sham group and the non-exercise group (P < 0.05), as indicated in Figure 2.

### 2.3. Behavioral Scores

**Figure 3.**
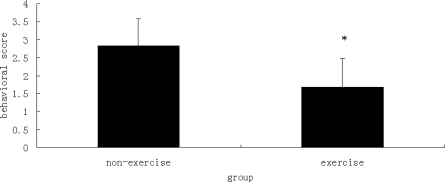
Behavioral scores of the exercise group and the non-exercise group. Data are mean ± S.D. (n = 6). The behavioral evaluation scores for the sham group were all zero. * indicates a statistically significant difference observed in the mean behavioral scores between the exercise group and the non-exercise group (P < 0.05).

Rats in the sham group, exercise with operation group and non-exercise with operation group, were evaluated 24 h after reperfusion. In the sham control group, none of the rats demonstrated neurological symptoms. In contrast, there was a significant difference in behavior scores between the exercise with operation and the non-exercise with operation groups (P < 0.05), as shown in Figure 3. The rats in the exercise with operation group showed fewer neurological deficits than those in the non-exercise with operation group.

### 2.4. Infarct Volume

**Figure 4.**
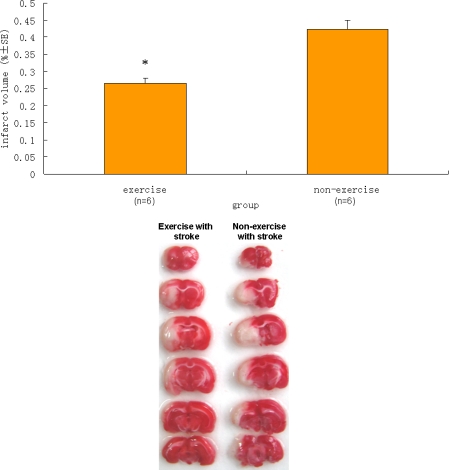
Difference in infarct volume between the exercise group and the non-exercise group. Top panel: Data are mean ± S.E. (n = 6). The infarct volume of the sham group of six rats was zero (not shown). ^*^ denotes a statistically significant difference in the mean infarct volumes of the exercise group and the non-exercise group (P < 0.05). Bottom panel: 2 mm coronal sections show ischemia (pale regions) and healthy tissue (red).

After behavioral evaluation, 18 rats were sacrificed to determine the infarct volume. None of the rats in the sham control group exhibited ischemic areas. In contrast, there was a significant difference in infarct volume between the exercise with operation and the non-exercise with operation groups (P < 0.05), as indicated in Figure 4. The rats in the exercise with operation group showed a significantly reduced ischemic area in the brain relative to those in the non-exercise with operation group.

### 2.5. Discussion

The glutamatergic system plays a key role in normal cerebral function and regulates the activity of glutamate, the most abundant neurotransmitter in the central nervous system (CNS), as well as ionotropic NMDA, AMPA and KA (kainic acid) receptors and metabotropic G protein-coupled receptors [16]. Furthermore, it is well known that glutamate signaling contributes to brain injury after stroke. Based on these findings, it has been suggested that targeting these glutamate receptor sub-types may serve as a novel therapeutic approach to treat stroke [17].

According to previous studies, group I *mGluRs* (including *mGluR1* and mGluR5) are especially important for the regulation of pyramidal cell excitability [18,19]. Furthermore, antagonists of group I mGluRs appear to be neuroprotective, while agonists can aggravate or alleviate neuronal cell death *in vitro* [20–22]. In addition, a previous study demonstrated that the selective *mGluR5* antagonist MPEP significantly reduced neuronal damage and improved neurological recovery in an animal model of focal cerebral ischemia [23]. The above observations are further supported by another report, which showed that MPEP and its structurally related selective *mGluR5* antagonist SIB-1893 significantly mitigated post-traumatic cell damage *in vitro* and improved functional recovery *in vivo* [24]. Another study demonstrated stronger neuroprotective effects of *mGluR5* antagonists compared to *mGluR1* antagonists [25]. Thus, inhibiting hyperactivation of *mGluR5* after ischemic stroke appears to be neuroprotective.

Other studies examining ionotropic NMDA receptors have implicated these ion channels in the induction of ischemic tolerance both *in vitro* and *in vivo*. Previous *in vivo* studies demonstrated that the NMDA receptor-dependent cellular signaling pathway contributes to the mechanism of ischemic tolerance and that new protein synthesis is also required in this process [26–28] In addition, a NMDA receptor non-competitive antagonist attenuated ischemic tolerance, whereas an AMPA receptor antagonist had no effect, thereby indicating that the NMDA receptor, but not the AMPA receptor, might be involved in the induction of ischemia tolerance [29].

*In vitro*, NMDA-type glutamate receptors have proved to be involved in both neuronal ischemic injury and ischemic tolerance [30]. Additionally, inhibiting *NR2B* attenuated ischemic cell death and strengthened preconditioning-induced neuroprotection [12].

Therefore, it seems that a drug that can influence NMDA-type glutamate receptors could be utilized to treat ischemic stroke. However, the development of NMDA receptor-specific drugs has been hindered by their serious side effects observed in clinical trials [31,32]. Thus, clarifying the molecular mechanisms by which glutamate receptors exert their functions in ischemic tolerance may offer a potential breakthrough for the development of new methods that prevent subsequent damage after stroke but have fewer side effects.

According to a previous study, both two-week and four-week pre-ischemic training could decrease the infarction volume and cerebral edema after MCAO [4]. Accordingly, it was reported that exercise preconditioning could reduce the level of inflammatory mediators and decrease the accumulation of leukocytes after cerebral occlusion/reperfusion [5]. Furthermore, pre-exercise also was able to alleviate cerebral permeability and strengthen brain microvascular integrity after MCAO [34].

In our previous study, we demonstrated that four weeks of treadmill training could down-regulate the excessive release of glutamate after ischemic stroke [14]. However, whether glutamate receptors are involved in this process is still unclear. According to a previous report, mGluR5 mRNA decreased significantly in ischemic preconditioning group compared to the non-preconditioning group [35]. In addition, in a previous *in vitro* study it was suggested that the mechanism of down-regulating NMDAR subunits mRNA levels by oxygen/glucose deprivation or glutamate receptor antagonists could be identical [36]. In the present study, the difference between the non-exercise group and sham group is whether the middle cerebral artery is occluded. Cerebral ischemia might influence expression of different gene at different time point. In our experiment, we detected the relative gene expression 80 min after occlusion. *NR2B* level significantly increased in the non-exercise group as compared with the sham group, but the similar result did not present in *mGluR5* level. It is speculated that at this time point the cerebral ischemia influences the relative gene expression of *NR2B*, but not *mGluR5*. On the other hand, according to previous reports [22–29], we may conclude that antagonism of *NR2B* and *mGluR5* could be neuroprotective in rats with MCAO. In our experiment, we showed that pre-training could reduce brain damage after cerebral ischemia. In addition, *NR2B* and *mGluR5* level significantly decreased in the exercise group compared to sham group or non-exercise group. It is conjectured that pre-training exerts neuroprotection via inhibiting the expression of *NR2B* and *mGluR5*. In addition, the exercise group exhibited fewer behavioral deficits and a reduced infarct volume relative to the non-exercise group. These findings indicate that both *NR2B* and *mGluR5* may contribute to the ischemic tolerance induced by treadmill training.

## 3. Experimental Methods

### 3.1. Animals

These experiments were performed according to the guidelines of the National Institutes of Health Guide for the Care and Use of Laboratory Animals. Thirty-six healthy male Sprague–Dawley rats (weighing 250–300 g) were provided by the Shanghai Laboratory Animal Center, Chinese Academy of Sciences. The rats were housed separately under a 12:12-h light/dark cycle with water and food available *ad libitum*.

### 3.2. Treadmill Training

Rats were randomly divided into three groups: a sham control group, an exercise with operation group and a non-exercise with operation group (12 rats in each group). The rats in the exercise group exercised at a speed of 6–9 m/min for 20 min per day to adapt to the running exercise. After that, the rats began formal training on an electric treadmill machine (DSPT-202 Type 5-Lane Treadmill; Litai Biotechnology Co., Ltd, China) over a period of four weeks. The formal training included a 25 m/min pace at a slope of 0° for 30 min/day, six days per week. The rats in the sham control group and non-exercise with operation group were housed freely in their cages for four weeks.

### 3.3. Rat MCAO Model

The protocol for our animal study was approved by the Animal Experimental Committee of Fudan, University at Shanghai, China. All rats were anesthetized intraperitoneally with chloral hydrate (10%) at a dose of 400 mg/kg. The body temperature was maintained by a heating pad. The rat model of middle cerebral artery occlusion (MCAO) was conducted according to the Zea *et al*. [33] with some modifications. Poly-*L*-lysine-coated intraluminal suture was used to guarantee the reliability of the stroke model. Such suture could yield larger infarction and significantly reduce inter-animal variability. First, we exposed the left common carotid artery (CCA), external carotid artery (ECA) and internal carotid artery (ICA). The ECA was ligated, and a small incision was made. After preparing a flame-rounded tip, a 20-mm long surgical suture was inserted into the internal carotid artery so as to occlude the middle cerebral artery, which was held to the ECA with a thread. In the sham control group, all steps were the same as in the operation group except for the occlusion of the middle cerebral artery. For half of the rats in each group, the suture was removed 2 h after occlusion. In order to measure physiological parameters, both 10 min before and after, MCAO blood samples were obtained from the femoral artery to detect pH, arterial partial pressure of oxygen (paO2) and carbon dioxide (paCO2) by a Blood Gas and Electrolyte System (Radiometer ABL505, Copenhagen, Denmark).

### 3.4. Evaluation of Behavioral Score

Half of the rats in each group were scored 24 h after reperfusion on a 5-point scale based on the report of Zea *et al*. [33] with the following criteria: 0, no neurological symptoms; 1, unable to completely extend the front jaw on the other side; 2, rotating while crawling and falling to the contralateral side; 3, unable to walk without help; and 4, unconsciousness.

### 3.5. Determination of Brain Infarction Volume

After evaluation for behavioral scores, animals were anesthetized with chloral hydrate (10%). The whole brain was removed as quickly as possible and placed at −20 °C for 10 min. The brain tissue was divided into six 2-mm stions in a coronal plane. The first stion was positioned at the middle line between the anterior pole and the optic chiasm. Afterwards, stions were simultaneously immersed in a 2% TTC (2,3,5-triphenyltetrazolium chloride) solution (37 °C) for 30 min, followed by a 4% paraformaldehyde buffer for fixation. After 24 h, all stions were photographed using a digital camera (DC240; Kodak, USA). Imaging software (Adobe Photoshop 7.0) was employed to evaluate the infarction volume (pink areas were normal and pale areas implied ischemia). The total infarction volume was calculated as the sum of the infarct areas from each stion. In order to minimize the error introduced by edema, we used an indirect method to calculate the infarct volumes [34]. Infarct volume = contralateral hemisphere region - non-infarcted region in the ipsilateral hemisphere Infarct percentage = Infarct volume/volume of the contralateral hemisphere × 100%

### 3.6. Tissue Processing

Half of the rats in each group were sacrificed 80 min after occlusion, and their brains were rapidly removed. The striatum was isolated and immediately frozen in liquid nitrogen until the next step.

### 3.7. Reverse Transcription

Stored tissue was homogenized with Trizol reagent (Invitrogen, Carlsbad, CA) for extraction of total RNA in accordance with the manufacturer’s protocol. Subsequently, a High Capacity cDNA Reverse Transcription Kit (Applied Biosystems) was used to complete the reverse transcription. Each tube contained a 20 μL reaction volume, which included 0.6 μg of total RNA (10 μL), 2 μL 10× RT buffer, 0.8 μL of 25× dNTP (100 mM), 2 μL 10× RT Random Primers and 50 units of MultiScribeTM Reverse Transcriptase. Tubes were heated for 10 min at 25 °C, 2 h at 37 °C and 5 s at 85 °C. The samples were stored at −20 °C until PCR amplification.

### 3.8. Quantitative Real-Time PCR

Gene-specific primers (sequences in Table 2) and SYBR® Green (Applied Biosystems, Foster City, CA) were used for real-time PCR quantification of NR2B and mGluR5 with an ABI PRISM 7000 real-time thermal cycler. Primer Express software (Applied Biosystems) was employed to design oligonucleotide primers. Glyceraldehyde-3-phosphate dehydrogenase (GAPDH) served as the standardized control.

The reaction settings were as follows: 1 cycle at 50 °C (2 min), 1 cycle at 95 °C (10 min) and 40 cycles of 95 °C for 15 s and 60 °C for 1 min. Cycle threshold values were selected based on the linear range from log scaling and the baseline. The 20 μL reaction contained 2 μL of cDNA, 300 nM of each primer and 10 μL of SYBR® Green PCR Master Mix, which included AmpliTaq Gold DNA polymerase. Sequence Detector software (SDS version 1.6, Applied Biosystems) was used for detecting SYBR Green reporter dye fluorescence. The relative change in the mRNA expression of the NR2B and mGluR5 following MCAO was calculated by the following equation [37]:

$$\text{Fold}\;{\text{change}=2}^{-\Delta \text{Ct}};\Delta {\text{Ct}=(\text{Ct}}_{\text{target}}-{\text{Ct}}_{\text{GAPDH}}).$$

### 3.9. Statistical Analysis

Statistical analyses were performed with SPSS for Windows, version 11.0 (SPSS Inc, Chicago, IL). Differences in relative mRNA expression among the groups were analyzed using a one-way ANOVA followed by a post-hoc Tamhane multiple comparison test to compare the three groups (Table 3). Statistical significance among behavioral scores and infarct volumes of the different experimental groups (exercise with occlusion and non-exercise with occlusion) was analyzed with an unpaired Student’s t-test. P < 0.05 was considered statistically significant.

## 4. Conclusions

Our study indicated that pre-ischemic treadmill training lowered the mRNA levels of both *NR2B* and *mGluR5*, which potentially induced BIT. Further study is needed to explore the possible involvement of other glutamate receptors and transporters in this neuroprotective effect. Overall, our study demonstrated that appropriate physical exercise may be an effective method for ischemic preconditioning.

## Figures and Tables

**Table 1.** Physiological variables among different groups before and after MCAO.

	**Before ischemia**	**After ischemia**
Sham		
pH	7.22 ± 0.01	7.23 ± 0.01
pCO2 (mmHg)	42.8 ± 3.5	43.1 ± 1.9
pO2 (mmHg)	103.8 ± 5.64	103.6 ± 2.5

Exercise		
pH	7.22 ± 0.02	7.22 ± 0.03
pCO2 (mmHg)	42.1 ± 2.2	42.5 ± 4.5
pO2 (mmHg)	102.9 ± 3.5	104.0 ± 3.3

Non-exercise		
pH	7.23 ± 0.04	7.22 ± 0.05
pCO2 (mmHg)	41.9 ± 3.1	42.6 ± 2.4
pO2 (mmHg)	102.8 ± 3.8	103.1 ± 5.7

No significant differences were observed in paO2, paCO2, or pH among the sham, non-exercise with stroke and exercise with stroke groups before and after MCAO. Data represent means ± standard error of the mean (S.E.M).

**Table 2.** Primer sequences used for real-time RT-PCR.

**Gene Forward primer (5′–3′)**	**Reverse primer (5′–3′)**
***NR2B:*** TCCGTCTTTCTTATGTGGATATGC CCTCTAGGCGGACAGATTAAGG	
***mGluR: 5*** VCCCTAAGCTCCAACGGAAAAT VTGATGACCGCCGTTTGGT	
***GAPDH:*** GGGCAGCCCAGAACATCA TGTCCGTATGGCTTCATTGATG	

**Table 3.** Relative gene expression levels of NR2B and mGluR5.

**Group**	**NR2B**	**mGluR5**
**Sham**	0.00182 ± 0.00026	0.01816 ± 0.00241
**Exercise**	0.00070 ± 0.00004	0.00286 ± 0.00061
**Non-exercise**	0.00335 ± 0.00076	0.01896 ± 0.00312

The different glutamate receptor subunits normalized with respect to the GAPDH mRNAs of each animal are demonstrated. Data represent means ± S.E.M. (n = 6).

## Abbreviations:

*mGluR5*: metabotropic glutamate receptor 5
*AMPA*: α-amino-3-hydroxi-5-methyl-4-isoxazol pro-pionic acid
*NMD*A: *N*-methyl-*d*-aspartate;
*NR2B*: NMDA receptor subunit type 2B
*MPEP*: 2-[^11^C]Methyl-6-(2-phenylethynyl)pyridine
*GAPDH*: glyceraldehyde-3-phosphate dehydrogenase
